# Die verschiedenen Phasen der COVID-19-Pandemie in Deutschland: Eine deskriptive Analyse von Januar 2020 bis Februar 2021

**DOI:** 10.1007/s00103-021-03394-x

**Published:** 2021-08-10

**Authors:** Julia Schilling, Kristin Tolksdorf, Adine Marquis, Mirko Faber, Thomas Pfoch, Silke Buda, Walter Haas, Ekkehard Schuler, Doris Altmann, Ulrike Grote, Michaela Diercke, RKI COVID-19 Study Group

**Affiliations:** 1 Abteilung für Infektionsepidemiologie, Robert Koch-Institut, Seestr. 10, 13353 Berlin, Deutschland; 2 HELIOS Kliniken GmbH, Berlin, Deutschland

**Keywords:** Pandemie, SARS-CoV‑2, Epidemiologie, Meldesystem, Syndromische Surveillance, Pandemics, SARS-CoV‑2, Epidemiology, Mandatory surveillance, Syndromic surveillance

## Abstract

Am 27.01.2020 wurde in Deutschland der erste Fall mit einer SARS-CoV-2-Infektion diagnostiziert. Für die Beschreibung des Pandemieverlaufs im Jahr 2020 wurden 4 epidemiologisch verschiedene Phasen betrachtet und Daten aus dem Meldesystem gemäß Infektionsschutzgesetz (IfSG) sowie hospitalisierte COVID-19-Fälle mit schwerer akuter respiratorischer Infektion aus der Krankenhaus-Surveillance eingeschlossen.

*Phase 0* umfasst den Zeitraum von Kalenderwoche (KW) 5/2020 bis 9/2020, in dem vor allem sporadische Fälle <60 Jahre und regional begrenzte Ausbrüche beobachtet wurden. Insgesamt wurden 167 Fälle übermittelt, die vorwiegend mild verliefen. Dem schloss sich in *Phase 1* (KW 10/2020 bis 20/2020) die erste COVID-19-Welle mit 175.013 Fällen im gesamten Bundesgebiet an. Hier wurden vermehrt Ausbrüche in Krankenhäusern, Alten- und Pflegeheimen sowie ein zunehmender Anteil an älteren und schwer erkrankten Personen verzeichnet. In *Phase 2*, dem „Sommerplateau“ mit eher milden Verläufen (KW 21/2020 bis 39/2020), wurden viele reiseassoziierte COVID-19-Fälle im Alter von 15–59 Jahren und einzelne größere, überregionale Ausbrüche in Betrieben beobachtet. Unter den 111.790 Fällen wurden schwere Verläufe seltener beobachtet als in *Phase 1*. *Phase 3* (KW 40/2020 bis 8/2021) war gekennzeichnet durch die zweite COVID-19-Welle in Deutschland, die sich zum Jahresende 2020 auf dem Höhepunkt befand. Mit 2.158.013 übermittelten COVID-19-Fällen und insgesamt deutlich mehr schweren Fällen in allen Altersgruppen verlief die zweite Welle schwerer als die erste Welle. Unabhängig von den 4 Phasen waren v. a. Ältere und auch Männer stärker von einem schweren Krankheitsverlauf betroffen.

## Abstract (en)

The first case of coronavirus SARS-CoV‑2 infection in Germany was diagnosed on 27 January 2020. To describe the pandemic course in 2020, we regarded four epidemiologically different periods and used data on COVID-19 cases from the mandatory reporting system as well as hospitalized COVID-19 cases with severe acute respiratory infection from the syndromic hospital surveillance.

*Period 0* covers weeks 5 to 9 of 2020, where mainly sporadic cases of younger age were observed and few regional outbreaks emerged. In total, 167 cases with mostly mild outcomes were reported. Subsequently, the first COVID-19 wave occurred in *period 1* (weeks 10 to 20 of 2020) with a total of 175,013 cases throughout Germany. Increasingly, outbreaks in hospitals and nursing homes were registered. Moreover, elderly cases and severe outcomes were observed more frequently. *Period 2* (weeks 21 to 39 of 2020) was an interim period with more mild cases, where many cases were younger and often travel-associated. Additionally, larger trans-regional outbreaks in business settings were reported. Among the 111,790 cases, severe outcomes were less frequent than in *period 1*. In *period 3* (week 40 of 2020 to week 8 of 2021), the second COVID-19 wave started and peaked at the end of 2020. With 2,158,013 reported cases and considerably more severe outcomes in all age groups, the second wave was substantially stronger than the first wave.

Irrespective of the different periods, more elderly persons and more men were affected by severe outcomes.

## Einleitung

Das neuartige Severe Acute Respiratory Syndrome Coronavirus 2 (SARS-CoV-2) wurde Ende Dezember 2019 erstmals in Wuhan, China entdeckt und breitete sich innerhalb weniger Wochen weltweit aus. Am 27.01.2020 wurde der erste Fall mit einer SARS-CoV-2-Infektion in Deutschland diagnostiziert [1], bis zum Mai 2021 wurde Deutschland dann von insgesamt 3 COVID-19-Wellen erfasst. Der folgende Artikel hat das Ziel, Daten aus verschiedenen Surveillance-Systemen zu kombinieren, um einen umfassenden Überblick zum Verlauf der COVID-19-Pandemie bis zum Ende der zweiten Welle zu geben. Hierbei werden sowohl die geografische Ausbreitung als auch die Demografie der Fälle und die Krankheitsschwere im zeitlichen Verlauf und im Kontext der infektionshygienischen Maßnahmen betrachtet. Zudem werden die beiden ersten COVID-19-Wellen in Deutschland verglichen.

## Methoden

Das COVID-19-Geschehen in Deutschland wird retrospektiv in 4 Phasen dargestellt:

- *Phase 0 (Auftreten sporadischer Fälle):* Kalenderwoche 5/2020 bis 9/2020,
- *Phase 1 (erste COVID-19-Welle):* Kalenderwoche 10/2020 bis 20/2020,
- *Phase 2 (Sommerplateau):* Kalenderwoche 21/2020 bis 39/2020
  (*Phase 2a*: 21/2020 bis 30/2020 und *Phase 2b*: 31/2020 bis 39/2020),
- *Phase 3 (zweite COVID-19-Welle):* Kalenderwoche 40/2020 bis 8/2021.

Die Abgrenzung der Phasen erfolgte anhand verschiedener epidemiologischer Parameter und wurde bereits an anderer Stelle ausführlich beschrieben [2]. Basierend auf diesem Vorgehen wurde das Ende der *Phase 3* (zweite Welle) in Kalenderwoche (KW) 8/2021 festgelegt.

Es wurden die an das Robert Koch-Institut (RKI) übermittelten Fälle gemäß Infektionsschutzgesetz (IfSG) entsprechend der RKI-Referenzdefinition [3] mit Datenstand 11.05.2021 betrachtet. In einer Analyse der Krankheitsschwere zur ersten COVID-19-Welle [4] hat sich herausgestellt, dass der Anteil intensivpflichtiger Fälle in den Meldedaten unterschätzt wird. Für ein vollständigeres Bild der kritischen Fälle wurden daher die ICD-10-code-basierten Daten zu hospitalisierten COVID-19-Fällen (COVID-SARI-Fälle) aus der Krankenhaus-Surveillance ICOSARI mit gleichem Datenstand herangezogen [5, 6].

### COVID-19-Fälle aus dem Meldesystem gemäß Infektionsschutzgesetz

Der direkte oder indirekte Nachweis von SARS-CoV‑2 sowie der Krankheitsverdacht, die Erkrankung und der Tod in Bezug auf eine Coronaviruskrankheit-2019 (COVID-19) sind nach Infektionsschutzgesetz (IfSG) meldepflichtig [3]. Die folgende Analyse basiert auf allen Fällen, die der Referenzdefinition (Nachweis von SARS-CoV‑2 mittels Polymerasekettenreaktion (PCR) oder Kultur, unabhängig von der Art der klinischen Symptomatik [3]) des RKI entsprechen und die von den Gesundheitsämtern über die Landesgesundheitsbehörden an das RKI übermittelt wurden. Es wurden alle Fälle mit einem Meldedatum zwischen Meldewoche (MW) 5/2020 und 8/2021 sowie einer Angabe zum Alter berücksichtigt. Gemäß § 11 IfSG müssen auch Krankheitshäufungen von COVID-19 an das RKI übermittelt werden. Je nach regionaler Ausbreitung werden Ausbrüche entweder im Gesundheitsamt oder auf Bundesland- bzw. Bundesebene zu einem größeren Ausbruchsgeschehen zusammengefasst. In der folgenden Auswertung wurde die Anzahl der Ausbrüche (mit mindestens 2 Fällen) auf der jeweils höchsten Ausbruchsebene betrachtet [7]. Als wahrscheinliche Infektionsumfelder der Ausbrüche wurden die häufigsten Ausbruchssettings angegeben [8].

### Meldungen aus dem Krankenhaus-Sentinel (ICOSARI)

Im Rahmen der seit 2015 bestehenden wissenschaftlichen Kooperation mit der HELIOS Kliniken GmbH hat das RKI ein kontinuierliches syndromisches Sentinel-Krankenhaus-Surveillance-System für schwere akute respiratorische Infektionskrankheiten (SARI) aufgebaut [5]. Das Sentinel umfasst dabei etwa 6 % aller Krankenhausfälle in Deutschland [9]. Im Rahmen dieses Systems werden anonymisiert Daten von stationär aufgenommenen Patienten erhoben (ICOSARI: ICD-10-code-basierte Krankenhaus-Surveillance schwerer akuter respiratorischer Infektionen). Dabei wurden COVID-SARI-Fälle definiert als Fälle mit einem Labornachweis für SARS-CoV‑2 (ICD-10-Code *U07.1!*: COVID-19, Virus nachgewiesen) sowie einer SARI (ICD-10-Codes *J09–J22*: Influenza sowie akute respiratorische Erkrankungen der unteren Atemwege). Für die deskriptive Analyse wurden COVID-SARI-Fälle betrachtet, die im Zeitraum von der Kalenderwoche (KW) 10/2020 bis zur KW 8/2021 in 72 Krankenhäusern des ICOSARI-Sentinels aufgenommen und von denen bis zum Datenstand 11.05.2021 eine Krankenhausentlassung, Verlegung oder ein Versterben übermittelt wurde. 7 Fälle, die zum Datenstand noch hospitalisiert waren, wurden von den Analysen ausgeschlossen.

### Deskriptive Analysen

Die folgende Auswertung fokussiert sich auf die geografische und demografische Verteilung sowie die Krankheitsschwere im zeitlichen Verlauf. Die Entwicklung wurde anhand der gemeldeten COVID-19-Fallzahlen gemäß IfSG nach Meldewoche (MW) dargestellt. Dies entspricht der Kalenderwoche, in der ein gemeldeter Fall beim Gesundheitsamt erfasst wurde. Die COVID-SARI-Fälle im ICOSARI-Sentinel wurden entsprechend der Kalenderwoche (KW) erfasst, in der sie stationär aufgenommen wurden.

Für die Beschreibung der verschiedenen Krankheitsverläufe wurden milde, moderate, schwere und kritische Verläufe und Todesfälle unterschieden (Tab. 1), wobei sich schwere und kritische Verläufe sowie Todesfälle nicht gegenseitig ausschließen. Diese Differenzierung basiert auf der initialen Verlaufsbeschreibung durch die Weltgesundheitsorganisation (WHO; [10]) und einer Bewertung der Krankheitsschwere basierend auf den Meldedaten gemäß IfSG [4, 11].

**Table 1:** 

Krankheitsverlauf	Meldesystem gemäß IfSG	Krankenhaus-Sentinel ICOSARI
*Mild*	Angaben zum klinischen Bild vorhanden, keine Pneumonie, nicht hospitalisiert, nicht verstorben	Nicht verfügbar
*Moderat*	Angaben zum klinischen Bild vorhanden, Pneumonie vorhanden, nicht hospitalisiert, nicht verstorben	Nicht verfügbar
*Schwer*	*Hospitalisiert* (unabhängig von klinischen Informationen, Intensivpflicht und Versterben)	*Hospitalisiert* (COVID-SARI-Fälle, definiert über ICD-10-Codes U07.1! + J09–J22)
*Kritisch*	nicht angewendet	*Intensivpflichtig* (COVID-SARI-Fälle mit Intensivbehandlung während der Hospitalisierung)
		*Beatmet* (COVID-SARI-Fälle mit mechanischer Beatmung während der Hospitalisierung)
*Verstorben*	*Verstorben* (unabhängig von klinischen Informationen und Hospitalisierung)	*Verstorben* (COVID-SARI-Fälle, die während ihrer Hospitalisierung verstorben sind)

*COVID-SARI* COVID-19-Fälle mit schwerer akuter respiratorische Infektion, *ICOSARI* ICD-10-code-basierte Krankenhaus-Surveillance schwerer akuter respiratorischer Infektionen, *IfSG* Infektionsschutzgesetz

Für die Auswertung der gemäß RKI-Referenzdefinition übermittelten Fälle in Bezug auf Krankheitsverlauf (mild, moderat) und Symptomatik wurden nur Fälle berücksichtigt, bei denen grundsätzlich eine Angabe zum klinischen Bild (Ja, Nein) vorlag.

Die Analyse wurde mithilfe von Microsoft Excel Professional 2019, QGIS Geographic Information System 3.14.1-Pi sowie StataSE Version 15 durchgeführt. Für die Berechnung von Inzidenzen wurde die Bevölkerungsstatistik mit Stand 31.12.2019 herangezogen [12].

## Ergebnisse

### Zeitliche und geografische Ausbreitung

**Figure 1:**
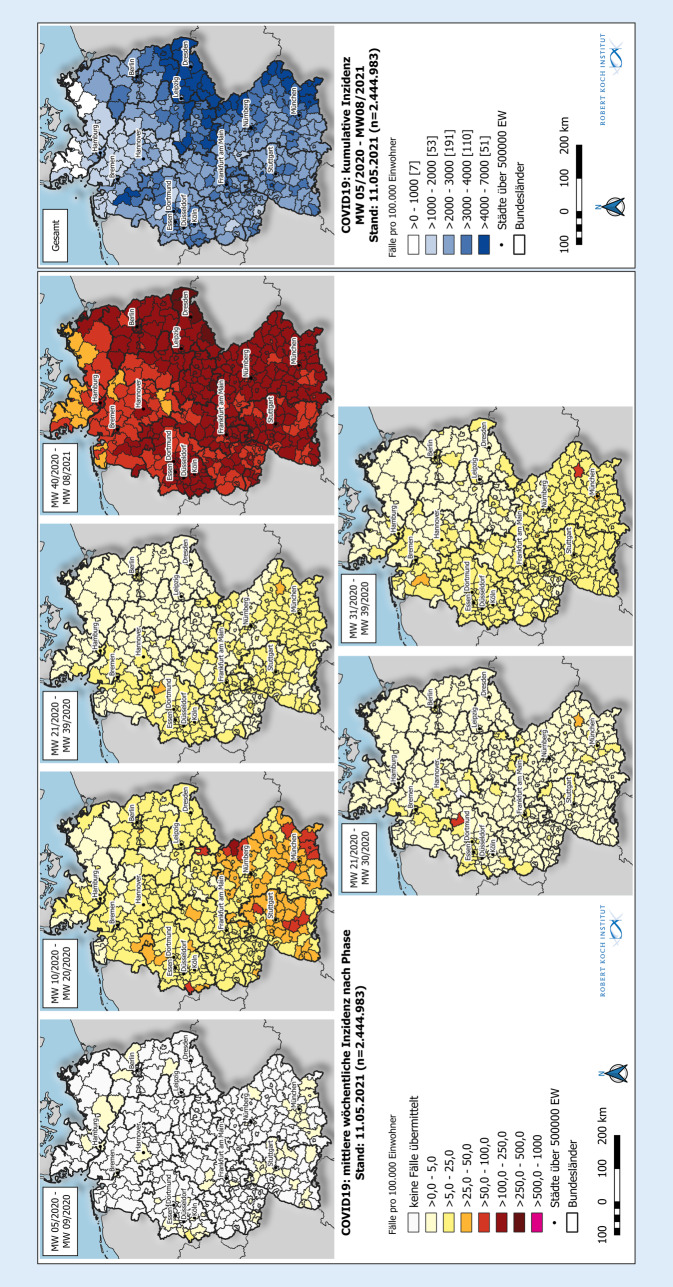

Zwischen MW 5/2020 und MW 8/2021 wurden insgesamt 2.444.983 COVID-19-Fälle übermittelt (Tab. 2). Im Zeitraum von MW 5/2020 bis 9/2020 (*Phase 0*) beschränkte sich das Infektionsgeschehen zunächst auf einzelne Stadt- und Landkreise (Abb. 1). Hierbei war die mittlere wöchentliche Inzidenz mit 5,8 pro 100.000 im Landkreis Heinsberg (Nordrhein-Westfalen) am höchsten.

**Table 2:** 

	Gesamt	Phase 0, sporadische Fälle	Phase 1 Erste Welle	Phase 2 Sommerplateau	Phase 3 Zweite Welle
*Gesamtzahl Fälle*	2.444.983	167	175.013	111.790	2.158.013
*Inzidenz (pro 100.000)*	2940	0,2	210	134	2595
*Mittlere wöchentl. Inz. (pro 100.000)*	52	0,04	19	7,1	118
*Höchste wöchentl. Inz. (pro 100.000)*	210 (MW 53/2020)	0,2 (MW 9/2020)	43 (MW 14/2020)	16 (MW 39/2020)	210 (MW 51/2020)
*Niedrigste wöchentl. Inz. (pro 100.000)*	0,01 (MW 5–8/2020)	0,01 (MW 5–8/2020)	1,08 (MW 10/2020)	2,8 (MW 24/2020)	19 (MW 40/2020)
*Altersgruppen (%, Inzidenz pro 100.000)*					
Altersmedian (Jahre)	44	36	50	33	45
0–4 Jahre	44.981 (1,8 %, 1135)	6 (3,6 %, 0,2)	1684 (1,0 %, 43)	3477 (3,1 %, 88)	39.814 (1,8 %, 1005)
5–14 Jahre	136.938 (5,6 %, 1843)	3 (1,8 %, 0,0)	3956 (2,3 %, 53)	9843 (8,8 %, 132)	123.136 (5,7 %, 1657)
15–34 Jahre	706.685 (29 %, 3696)	71 (43 %, 0,4)	43.719 (25 %, 229)	46.168 (41 %, 241)	616.727 (29 %, 3226)
35–59 Jahre	930.393 (38 %, 3217)	67 (40 %, 0,2)	72.908 (42 %, 252)	39.328 (35 %, 136)	818.090 (38 %, 2829)
60–79 Jahre	378.120 (15 %, 2094)	19 (11 %, 0,1)	32.968 (19 %, 183)	9517 (8,5 %, 53)	335.616 (16 %, 1859)
≥80 Jahre	247.866 (10 %, 4363)	1 (0,6 %, 0,0)	19.778 (11 %, 348)	3457 (3,1 %, 61)	224.630 (10 %, 3954)
*Geschlecht (%, Inzidenz pro 100.000)*					
Weiblich	1.283.441 (52 %, 3046)	80 (48 %, 0,2)	91.449 (52 %, 217)	52.568 (47 %, 125)	1.139.344 (53 %, 2704)
Männlich	1.145.985 (47 %, 2793)	87 (52 %, 0,2)	83.416 (48 %, 203)	58.793 (53 %, 143)	1.003.689 (47 %, 2446)
Unbekannt	15.557 (0,6 %)	–	148 (0,1 %)	429 (0,4 %)	14.980 (0,7 %)
*Expositionsort*					
Deutschland	1.413.412 (58 %)	117 (70 %)	101.328 (58 %)	56.759 (51 %)	1.255.208 (58 %)
Ausland	63.682 (2,6 %)	26 (16 %)	16.281 (9,3 %)	25.880 (23 %)	21.495 (1,0 %)
Unbekannt	967.889 (40 %)	24 (14 %)	57.404 (33 %)	29.151 (26 %)	881.310 (41 %)
*Ausbrüche*					
Ausbruchsfälle (%)	499.722 (20 %)	116 (70 %)	43.338 (25 %)	37.328 (33 %)	418.940 (19 %)
Altersmedian (Ausbruchsfälle)	50	37	53	32	52
Mittlere wöchentl. Anzahl Ausbrüche	1420	3,6	571	382	3063
Häufigste Ausbruchssettings	Privater Haushalt, Alten- und Pflegeheime, Arbeitsplatz	Privater Haushalt, Arbeitsplatz, Hotel/Herberge/Pension	Privater Haushalt, Alten- und Pflegeheime, Krankenhäuser, Arbeitsplatz	Privater Haushalt, Arbeitsplatz, Freizeit	Privater Haushalt, Alten- und Pflegeheime, Arbeitsplatz

Anteile unter 10 % werden mit einer Kommastelle angegeben

**Figure 2:**
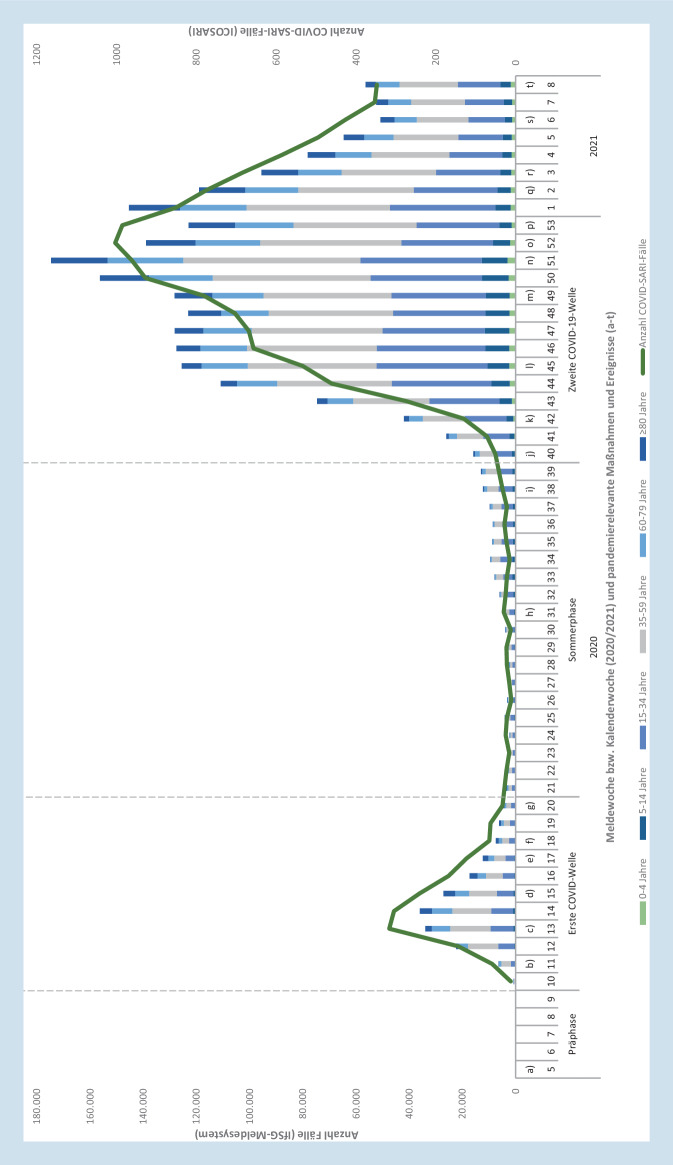

Ab MW 10/2020 folgte dann die erste COVID-19-Welle in Deutschland (*Phase 1*, Abb. 2). Ab MW 11/2020 wurden erstmals aus allen Bundesländern Fälle berichtet. Zur gleichen Zeit wurden die infektionshygienischen Maßnahmen zur Kontaktbeschränkung verschärft und zu einem umfassenden Lockdown in KW 13/2020 ausgeweitet. Insbesondere die südlichen Landkreise in Bayern und Baden-Württemberg waren von hohen Fallzahlen betroffen (Abb. 1). Bundesweit wurde der Höchstwert der wöchentlichen Inzidenz in MW 14/2020 mit 43 pro 100.000 erreicht (Tab. 2). Mit dem Ende der ersten COVID-19-Welle in MW 20/2020 wurden die kontaktbeschränkenden Maßnahmen weiter sukzessive gelockert (Abb. 2).

Im Zeitraum von MW 21/2020 bis 39/2020 (*Phase 2*) nahmen die Fallzahlen zunächst weiterhin kontinuierlich ab und sanken in MW 24/2020 auf die niedrigste wöchentliche Inzidenz von 2,8 pro 100.000 (Abb. 2; Tab. 2). Ab Mitte der *Phase 2* kam es zu einem kontinuierlichen Anstieg der Fallzahlen. Auffällig waren hierbei die Landkreise Gütersloh (mittlere wöchentliche Inzidenz 35 pro 100.000) und Dingolfing-Landau (mittlere wöchentliche Inzidenz 42 pro 100.000; Abb. 1). Parallel dazu wurden ab MW 31/2020 kostenlose Tests für aus dem Ausland Einreisende ermöglicht (Abb. 2).

Mit Beginn des Herbstes kam es ab MW 40/2020 zu einem sprunghaften Anstieg der wöchentlichen Fallzahlen (*Phase 3*, Abb. 2). Kurze Zeit später – in MW 42/2020 – folgte eine Anpassung der Teststrategie und damit eine Ausweitung der Testungen auf Antigen-Point-of-Care-Tests in Alten- und Pflegeheimen und Krankenhäusern. Dem weiteren Anstieg der Fallzahlen folgten in MW 45/2020 ein Teillockdown mit weiteren Kontaktbeschränkungen und ab Dezember 2020 weitere Verschärfungen und Anpassungen der Teststrategie. Nach dem Höhepunkt der zweiten Welle in MW 51/2020 mit einer wöchentlichen Inzidenz von 210 pro 100.000 sanken die Fallzahlen ab MW 1/2021 kontinuierlich bis MW 6/2021 und stagnierten bis MW 8/2021 auf hohem Niveau (Tab. 2). Wenige Kreise (5; 1,2 %) überschritten eine mittlere wöchentliche Inzidenz von 250 pro 100.000 (Abb. 2). Dies waren insbesondere Kreise im Raum Sachsen an der Grenze zur Tschechischen Republik. Bundesweit lag die mittlere wöchentliche Inzidenz in dieser Phase bei 118 Fällen pro 100.000 (Tab. 2).

### Expositionsorte und Ausbrüche

Der Anteil der Fälle mit einer wahrscheinlichen Exposition im Ausland war vor allem in *Phase 0 *(16 %) und *Phase 2* (23 %) erhöht und nahm während der COVID-19-Wellen in *Phase 1* (9,3 %) und *Phase 3* (1,0 %) deutlich ab (Tab. 2).

Über alle Phasen hinweg wurden vor allem Ausbrüche im privaten Haushalt und am Arbeitsplatz berichtet. Wurden zu Beginn der Pandemie in Deutschland (*Phase 0*) neben diesen noch Übernachtungsmöglichkeiten (z. B. Hotel, Herberge, Pension) als häufigstes Ausbruchssetting angegeben, verschob sich der Schwerpunkt in der ersten COVID-19-Welle zunehmend in Alten- und Pflegeheime sowie Krankenhäuser. Mit Beginn der Urlaubs- und Ferienzeit in *Phase 2* wurden auch Freizeitaktivitäten als häufigste Ausbruchssettings genannt. In *Phase 3* waren neben dem privaten Haushalt erneut Alten- und Pflegeheime und der Arbeitsplatz häufige Ausbruchssettings. Der Altersmedian von Fällen in Ausbrüchen lag in den *Phasen 0 und 2* deutlich niedriger (37 bzw. 32 Jahre) als während der beiden COVID-19-Wellen in den *Phasen 1 und 3* (53 bzw. 52 Jahre).

### Krankheitsschwere

#### Demografie

Der Anteil weiblicher und männlicher Fälle war gleich verteilt und variierte im Verlauf des Jahres nur um wenige Prozentpunkte (Tab. 2). Insgesamt wurden vor allem COVID-19-Fälle im Alter von 35 bis 59 Jahren übermittelt (38 %). Diese Altersgruppe machte in jeder Phase den größten Anteil unter den übermittelten Fällen aus. Mit einer altersspezifischen Inzidenz von 4363 pro 100.000 im gesamten Beobachtungszeitraum wurde jedoch die Altersgruppe der Personen ab 80 Jahren am häufigsten diagnostiziert. Mit Blick auf die Phasen war insbesondere während der COVID-19-Wellen (*Phase 1 und 3*) in dieser Altersgruppe die höchste Inzidenz zu beobachten. In den Interimsphasen (*Phase 0 und 2*) wurde dagegen die Altersgruppe der 15- bis 34-Jährigen häufiger diagnostiziert. Der höchste Altersmedian lag mit 50 Jahren in *Phase 1*, der niedrigste mit 33 Jahren in *Phase 2*.

#### Klinik

Die 3 häufigsten übermittelten Symptome waren Allgemeinbeschwerden (z. B. Schwäche, allgemeines Krankheitsgefühl, Gliederschmerzen; 49 %), Husten (41 %) und Fieber (29 %). In der Altersgruppe 0 bis 4 Jahre wurde am häufigsten Fieber (35 %) angegeben, wohingegen in den übrigen Altersgruppen Allgemeinbeschwerden am häufigsten genannt wurden (Tab. 3). Unter allen Fällen mit übermittelten Symptomen wurden im Median 2 Symptome je Fall angegeben, jedoch variierte die Anzahl der genannten Symptome zwischen den Altersgruppen. So wurden für Kinder und Hochaltrige eher keine Symptome angegeben. Unter Fällen mit nur einem Symptom wurden vor allem Allgemeinbeschwerden genannt. Darüber hinaus traten bei Kindern (0–4 und 5–14 Jahre) vor allem Fieber und Schnupfen als einziges Symptom auf. Unter den Erwachsenen (15–59 Jahre) wurden dagegen eher Husten und Schnupfen bzw. bei Senioren (ab 60 Jahre) eher Husten und Fieber genannt.

**Table 3:** 

Altersgruppen	Gesamt		0–4 Jahre		5–14 Jahre		15–34 Jahre		35–59 Jahre		60–79 Jahre		≥80 Jahre	
**Anzahl Fälle (*n*)**	2.444.983		44.981		136.938		706.885		930.393		378.120		247.866	
**Anzahl Nennungen**	***n***	**%**	***n***	**%**	***n***	**%**	***n***	**%**	***n***	**%**	***n***	**%**	***n***	**%**
*Klinische Informationen vorhanden*														
Ja	1.733.666	71	29.590	66	89.737	66	511.851	72	689.379	74	267.312	71	145.800	59
Nein	226.653	9,3	6542	15	20.380	15	57.166	8,1	70.774	7,6	36.358	9,6	35.433	14
Unbekannt	484.664	20	8849	20	26.821	20	137.668	20	170.243	18	74.450	20	66.633	27
*Symptome*														
Allgemeine Krankheitszeichen	851.705	49	5944	20	27.701	31	256.840	50	377.356	55	130.493	49	53.371	37
Husten	702.233	41	8417	29	20.162	23	207.718	41	309.973	45	115.416	43	40.547	28
Schnupfen	502.699	29	7882	27	21.535	24	180.860	35	216.747	31	60.091	23	15.584	11
Fieber	463.364	27	10.222	35	18.815	21	128.770	25	200.259	29	73.950	28	31.348	22
Halsschmerzen	370.516	21	2032	6,9	14.208	16	139.823	27	160.797	23	42.677	16	10.979	7,5
Geschmacksverlust	308.273	18	499	1,7	6132	6,8	132.323	26	137.579	20	27.323	10	4417	3,0
Geruchsverlust	280.860	16	363	1,2	5277	5,9	121.269	24	127.206	19	23.618	8,8	3127	2,1
Dyspnoe	83.993	4,8	305	1,0	555	0,6	17.433	3,4	31.853	4,6	19.153	7,2	14.694	10
Durchfall	80.956	4,7	1508	5,1	2474	2,8	19.826	3,9	34.596	5,0	15.889	5,9	6663	4,6
Pneumonie	26.486	1,5	53	0,9	91	0,1	1447	0,3	5353	0,8	8836	3,3	10.706	7,3
ARDS^a^	14.415	0,8	42	0,1	110	0,1	2258	0,4	4500	0,7	4329	1,6	3176	2,2
Tachykardie	5410	0,3	14	0,1	40	0,04	1153	0,2	2221	0,3	1199	0,5	783	0,5
Tachypnoe	5846	0,3	23	0,1	42	0,1	1388	0,3	2443	0,4	1188	0,4	762	0,5
*Anzahl Symptome*														
1 Symptom	334.209	19	7969	27	21.653	24	84.825	17	125.333	18	57.423	22	37.006	25
2 Symptome	397.990	23	6458	22	18.821	21	115.682	23	164.360	24	64.286	24	28.383	20
3 Symptome	324.692	19	3320	11	10.220	11	103.827	20	141.612	21	48.535	18	17.178	12
Mind. 4 Symptome	338.080	20	1493	5,1	6058	6,8	122.845	24	154.298	22	41.847	16	11.539	7,9
Unbekannt	338.695	20	10.350	35	32.985	37	84.672	17	103.773	15	55.221	21	51.694	36

Anteile unter 10 % werden mit einer Kommastelle angegeben

^a^*ARDS* Acute Respiratory Distress Syndrom

#### Krankheitsverläufe

Die Krankheitsverläufe werden im Folgenden basierend auf den übermittelten Fällen gemäß IfSG sowie der Krankenhaus-Surveillance ICOSARI beschrieben.

Insgesamt waren laut *Meldesystem gemäß IfSG* 1.337.428 Fälle (77 %) mild erkrankt, 192.191 Fälle (10 %) stationär aufgenommen worden und 75.402 Fälle (3,1 %) verstorben (Tab. 4). Mit zunehmendem Alter stieg der Anteil an hospitalisierten und verstorbenen Fällen. So war die Mehrheit der Fälle unter 60 Jahren nur mild erkrankt. Dagegen hatte weniger als die Hälfte der Fälle ab 80 Jahren einen milden Verlauf und es wurde in dieser Altersgruppe der höchste Anteil hospitalisierter bzw. verstorbener Fälle beobachtet. Entsprechend lag der Altersmedian bei milden Fällen deutlich niedriger als bei hospitalisierten und verstorbenen Fällen (41 Jahre vs. 73 bzw. 84 Jahre). Im Vergleich zwischen der ersten und zweiten Welle (*Phase 1 und 3*) gab es hier nur wenige Unterschiede. In *Phase 2* (Sommerplateau) war der Altersmedian hospitalisierter Fälle jedoch mit 58 Jahren deutlich niedriger.

**Table 4:** 

	Gesamt			0–4 Jahre		5–14 Jahre		15–34 Jahre		35–59 Jahre		60–79 Jahre		≥80 Jahre	
Verlauf	Altersmedian	*n*	%	*n*	%	*n*	%	*n*	%	*n*	%	*n*	%	*n*	%
*Gesamt (2020)*															
Mild	**41**	1.337.428	77	25.061	85	77.604	87	434.572	85	559.744	81	175.276	66	65.171	45
Moderat	**49**	3388	0,2	26	0,1	64	0,1	809	0,2	1532	0,2	704	0,3	253	0,2
Hospitalisiert	**73**	192.191	10	1402	4,0	1190	1,1	13.006	2,4	39.299	5,6	68.360	23	68.934	36
Verstorben	**84**	75.402	3,1	5	0	5	0	99	0	2503	0,3	20.566	5,5	52.224	21
*Phase 0, sporadische Fälle*															
Mild	**33**	97	65	2	^a^	3	^a^	48	74	33	55	10	^a^	1	^a^
Moderat	**k.** **A.**	0	0	0	^a^	0	^a^	0	0	0	0	0	^a^	0	^a^
Hospitalisiert	**40,5**	48	32	3	^a^	3	^a^	16	25	24	41	5	^a^	0	^a^
Verstorben	**65**	1	0,6	0	^a^	0	^a^	0	0	0	0	1	^a^	0	^a^
*Phase 1, erste Welle*															
Mild	**46**	111.188	72	1105	80	2810	86	32.545	85	52.412	80	16.544	57	5772	36
Moderat	**52**	438	0,3	1	0,1	3	0,1	81	0,2	224	0,3	98	0,3	31	0,2
Hospitalisiert	**71**	27.729	18	164	11	112	3,3	1684	4,5	6491	10	10.691	36	8587	48
Verstorben	**82**	8912	5,1	1	0,1	0	0,0	19	0,0	406	0,6	2891	8,8	5595	28
*Phase 2, Sommerplateau*															
Mild	**31**	70.973	82	2202	87	6205	89	31.381	87	25.138	82	4990	66	1057	42
Moderat	**42**	169	0,2	0	0	8	0,1	54	0,1	70	0,2	34	0,4	3	0,1
Hospitalisiert	**58**	7648	8,0	122	4,1	121	1,4	1141	2,9	2586	7,7	2212	27	1466	47
Verstorben	**81**	864	0,8	0	0	0	0	5	0	74	0,2	295	3,2	490	17
*Phase 3, zweite Welle*															
Mild	**41**	1.155.170	77	21.752	85	68.586	86	370.598	85	482.161	81	153.732	67	58.341	46
Moderat	**49**	2781	0,2	25	0,1	53	0,1	674	0,2	1238	0,2	572	0,2	219	0,2
Hospitalisiert	**74**	156.766	9,7	1113	3,6	957	1,0	10.165	2,2	30.198	5,0	55.452	22	58.881	35
Verstorben	**84**	65.625	3,1	4	0	5	0	75	0	2023	0,2	17.379	5,2	46.139	21

Anteile unter 10 % werden mit einer Kommastelle angegeben

*n* gibt die Anzahl der Fälle zum jeweiligen Krankheitsverlauf an; der Anteil bezieht sich auf die Gesamtzahl der Fälle, zu denen eine Angabe zum jeweiligen Krankheitsverlauf (Ja, Nein) vorhanden war

^a^Bei einer Gesamtzahl (hier Fälle mit vorhandener Angabe) von weniger als 20 Fällen wird kein Anteil ausgewiesen

In allen Altersgruppen wurde in *Phase 1* der höchste Anteil an hospitalisierten und verstorbenen Fällen beobachtet. Die Mehrzahl der hospitalisierten Fälle in *Phase 1* war im Alter von 60 bis 79 Jahren. Im Vergleich dazu war die Mehrzahl der Fälle in der zweiten Welle (*Phase 3*) 80 Jahre oder älter. Unter verstorbenen Fällen wurden in allen Phasen am häufigsten Personen ab 80 Jahren übermittelt.

Im Rahmen der *Krankenhaus-Surveillance ICOSARI* wurden insgesamt 14.703 COVID-SARI-Fälle stationär aufgenommen, im Median 76 Jahre alt und 9 Tage hospitalisiert. Während der Altersmedian bei COVID-SARI-Fällen in *Phase 2* deutlich niedriger lag als in den *Phasen 1 und 3*, war die mediane Hospitalisierungsdauer in den verschiedenen Phasen ähnlich (Tab. 5). Unter diesen Fällen wurden 8368 (57 %) Fälle bis zum 11.05.2021 nach Hause entlassen und weitere 2583 (18 %) COVID-SARI-Fälle in eine andere medizinische Einrichtung verlegt.

**Table 5:** 

	Gesamt			Phase 1			Phase 2			Phase 3		
	*n*	% an hospitalisiert	Median	*n*	% an hospitalisiert	Median	*n*	% an hospitalisiert	Median	*n*	% an hospitalisiert	Median
**Hospitalisiert**												
*Alter (Jahre)*	–	–	76	–	–	73	–	–	59	–	–	76
*Dauer (Tage)*^b^	–	–	9	–	–	10	–	–	9	–	–	9
0–4 Jahre	28	100 %	–	1	^a^	–	4	^a^	–	23	100 %	–
5–14 Jahre	9	^a^	–	1	^a^	–	2	^a^	–	6	^a^	–
15–34 Jahre	432	100 %	–	52	100 %	–	46	100 %	–	334	100 %	–
35–59 Jahre	2810	100 %	–	358	100 %	–	168	100 %	–	2284	100 %	–
60–79 Jahre	5694	100 %	–	615	100 %	–	137	100 %	–	4942	100 %	–
≥80 Jahre	5730	100 %	–	504	100 %	–	81	100 %	–	5145	100 %	–
Gesamt	14.703	100 %	–	1531	100 %	–	438	100 %	–	12.734	100 %	–
**Intensivpflichtig**												
*Alter (Jahre)*	–	–	73	–	–	72	–	–	70	–	–	74
*Dauer (Tage)*^b^	–	–	15	–	–	16	–	–	18,5	–	–	15
0–4 Jahre	2	7 %	–	0	^a^	–	2	^a^	–	0	0 %	–
5–14 Jahre	2	^a^	–	0	^a^	–	1	^a^	–	1	^a^	–
15–34 Jahre	66	15 %	–	8	15 %	–	3	7 %	–	55	16 %	–
35–59 Jahre	850	30 %	–	120	34 %	–	28	17 %	–	702	31 %	–
60–79 Jahre	2326	41 %	–	289	47 %	–	59	43 %	–	1978	40 %	–
≥80 Jahre	1535	27 %	–	162	32 %	–	29	36 %	–	1344	26 %	–
Gesamt	4781	33 %	–	579	38 %	–	122	28 %	–	4080	32 %	–
**Mechanisch beatmet**												
*Alter (Jahre)*	–	–	72	–	–	71	–	–	72	–	–	72
*Dauer (Tage)*^b^	–	–	17	–	–	20	–	–	22	–	–	16
0–4 Jahre	2	7 %	–	0	^a^	–	2	^a^	–	0	0 %	–
5–14 Jahre	0	^a^	–	0	^a^	–	0	^a^	–	0	^a^	–
15–34 Jahre	31	7 %	–	5	10 %	–	0	0 %	–	26	8 %	–
35–59 Jahre	536	19 %	–	67	19 %	–	14	8 %	–	455	20 %	–
60–79 Jahre	1611	28 %	–	192	31 %	–	41	30 %	–	1378	28 %	–
≥80 Jahre	761	13 %	–	74	15 %	–	18	22 %	–	669	13 %	–
Gesamt	2941	20 %	–	338	22 %	–	75	17 %	–	2528	20 %	–
**Verstorben**												
*Alter (Jahre)*	–	–	82	–	–	81	–	–	80	–	–	82
*Dauer (Tage)*^b^	–	–	10	–	–	10	–	–	13	–	–	10
0–4 Jahre	0	0 %	–	0	^a^	–	0	^a^	–	0	0 %	–
5–14 Jahre	0	^a^	–	0	^a^	–	0	^a^	–	0	^a^	–
15–34 Jahre	4	1 %	–	2	4 %	–	0	0 %	–	2	1 %	–
35–59 Jahre	139	5 %	–	14	4 %	–	1	1 %	–	124	5 %	–
60–79 Jahre	1304	23 %	–	128	21 %	–	18	13 %	–	1158	23 %	–
≥80 Jahre	2305	40 %	–	185	37 %	–	26	32 %	–	2094	41 %	–
Gesamt	3752	26 %	–	329	21 %	–	45	10 %	–	3378	27 %	–

Anteile unter 10 % werden mit einer Kommastelle angegeben

^a^Bei einer Gesamtzahl von weniger als 20 Fällen wird kein Anteil ausgewiesen

^b^Dauer der Hospitalisierung in Tagen

Mit 54 % war die Mehrheit der Fälle männlich – insbesondere unter den intensivpflichtigen (62 %), beatmeten (65 %) und stationär verstorbenen Fällen (58 %). Das Geschlechterverhältnis je nach Krankheitsverlauf veränderte sich im Jahresverlauf nur um wenige Prozentpunkte.

Insgesamt wurden 4781 (33 %) Personen intensivmedizinisch behandelt und im Median 15 Tage hospitalisiert (Tab. 5). In *Phase 2* wurden sie jedoch etwas länger stationär behandelt und waren im Median etwas jünger als in den *Phasen 1 und 3*. Darüber hinaus war in *Phase 2* der Anteil der Intensivbehandlungen bei COVID-SARI-Fällen am niedrigsten. Beim Vergleich der Wellen lag der Anteil der intensivpflichtigen Fälle während der zweiten Welle (*Phase 3*) niedriger als während der ersten Welle (*Phase 1*), insbesondere in den Altersgruppen ab 60 Jahre.

Während ihrer stationären Behandlung wurden 2941 (20 %) COVID-SARI-Fälle mechanisch beatmet (Tab. 5). Im Median waren diese Fälle 73 Jahre alt, hier gab es kaum Unterschiede zwischen den einzelnen Phasen. Beatmete Fälle waren insbesondere in den *Phasen 1 und 2* im Median deutlich länger hospitalisiert als intensivpflichtige COVID-SARI-Fälle insgesamt. In *Phase 3* waren mechanisch beatmete Patienten im Median dagegen kaum länger hospitalisiert als intensivpflichtige COVID-SARI-Fälle. Hierbei zeigte sich vor allem ein Unterschied bei beatmeten Patienten mit einer späteren Verlegung in ein anderes Krankenhaus: Wurden diese Patienten in der ersten Welle im Median nach 27 Tagen verlegt, so erfolgte die Verlegung in der zweiten Welle bereits im Median nach 18 Tagen. Beatmungspflichtige Patienten ohne Verlegung waren dagegen in beiden Wellen ähnlich lange hospitalisiert (17 bzw. 16 Tage).

Während ihres Krankenhausaufenthalts verstarben 3752 (26 %) der COVID-SARI-Fälle (Tab. 5). Hierbei lag der Altersmedian bei 82 Jahren und damit deutlich über dem Median der COVID-SARI-Patienten insgesamt. Während der beiden Wellen verstarben auch mehrere COVID-SARI-Fälle aus der Altersgruppe 35 bis 59 Jahre (4 % bzw. 5 %) im Krankenhaus. In der zweiten Welle wurde insgesamt ein höherer Anteil Todesfälle berichtet als in der ersten Welle. In *Phase 2* gab es den niedrigsten Anteil an Todesfällen unter den COVID-SARI-Fällen, jedoch wurden sie in dieser Phase im Median etwas länger hospitalisiert.

## Diskussion

Insgesamt war das COVID-19-Infektionsgeschehen in Deutschland durch unterschiedliche Phasen geprägt, die durch 2 unterschiedlich schwer verlaufende COVID-19-Wellen im Frühjahr und Herbst und einer Sommerplateauphase mit eher milden Verläufen charakterisiert waren.

Der erste laborbestätigte Fall mit einer SARS-CoV-2-Infektion wurde in Deutschland in MW 5/2020 übermittelt [1, 13, 14]. Innerhalb der folgenden Wochen wurden weitere Fälle mit einer Exposition im Ausland bekannt. Darunter befanden sich aus China repatriierte Personen [14, 15], aber auch Einreisende aus Urlaubsgebieten (u. a. Skigebiete in Italien und Österreich; [16, 17]). Sie spielten zum Teil eine tragende Rolle für das Infektionsgeschehen auf regionaler Ebene [16], was den vergleichsweise hohen Anteil importierter Fälle zu Beginn der Pandemie erklärt. Mitte Februar folgten Ausbrüche in Zusammenhang mit lokalen Feiern (z. B. Karneval), die zu erhöhten Fallzahlen in einzelnen Kreisen führten (u. a. LK Heinsberg, LK Tirschenreuth; [15, 16, 18, 19]) und den Beginn der ersten COVID-19-Welle einleiteten.

In der *Phase 1* waren alle Bundesländer betroffen, wobei lokale Feiern und die Nähe zu Grenzregionen mit einem erhöhten Infektionsgeschehen eine wichtige Rolle für regionale Ausbreitung spielten [14, 20]. Ausbruchsgeschehen traten nun auch vermehrt in stationären Einrichtungen auf. Laut Buda et al. sind stationäre Ausbruchssettings mit einer höheren Anzahl an Fällen assoziiert [8], was sich auch im zunehmenden Anteil an Fällen höheren Alters in *Phase 1* widerspiegelte.

Mit dem Ende der ersten Welle begann die *Phase 2*, in der alle Bundesländer nur sehr niedrige Inzidenzen meldeten. Insgesamt gab es in *Phase 2* nur wenige Vorgaben für kontaktreduzierende Maßnahmen. Dagegen wurden jedoch zusätzliche, niedrigschwellige und kostenlose Testmöglichkeiten für aus dem Ausland Einreisende ab KW 31/2020 geschaffen, welche erst mit dem Ende der Sommerferien in allen Bundesländern in KW 38/2020 für Einreisende aus Nichtrisikogebieten eingestellt wurden [21]. Die zunehmende Reisetätigkeit in den Sommermonaten und die zunehmend zur Verfügung stehenden Testmöglichkeiten spiegelten sich u. a. im Anstieg von Fällen mit einer wahrscheinlichen Exposition im Ausland und der Anzahl an Ausbrüchen im Freizeitbereich wider. Im Vergleich zu den anderen Phasen wurde in *Phase 2* der jüngste Altersmedian (33 Jahre) unter den übermittelten COVID-19-Fällen verzeichnet. Dies ist wahrscheinlich darauf zurückzuführen, dass diese reiseassoziierten Fälle vergleichsweise jünger waren und durch die sensitive Teststrategie an den Flughäfen im Sommer (im Vergleich zur *Phase 0*) auch (ebenfalls vermehrt junge) asymptomatische Fälle erkannt wurden [22]. Auch die großen Ausbrüche in Betrieben mit überwiegend jungen Beschäftigten (u. a. in Fleisch verarbeitenden Betrieben, wie bspw. in Gütersloh, oder unter Erntehelfern, wie bspw. im Kreis Dingolfing-Landau) trugen hierzu bei [14, 23, 24].

Die *Phase 3* war insbesondere geprägt durch autochthone Fälle sowie eine sechsmal höhere Anzahl an Ausbrüchen im Wochenmittel als in der ersten Welle. Wurden zu Beginn der Phase eher Fälle unter 60 Jahren diagnostiziert, nahmen im weiteren Verlauf mit der Anzahl an Ausbrüchen in stationären Einrichtungen auch die Fälle unter älteren Personen und damit auch unter Personen mit hohem Risiko für einen schweren Verlauf zu. Der hohe Anteil an unbekannten Expositionsorten und Ausbruchssettings spricht zudem für ein zunehmend diffuses Infektionsgeschehen, in dem Infektionsketten nur noch unzureichend ermittelt werden konnten.

Mit Blick auf die Krankheitsverläufe war der Anteil hospitalisierter Fälle mit 32 % in *Phase 0* am höchsten. Jedoch wurden in dieser Phase bestätigte COVID-19-Fälle zum Zweck der Beobachtung im Krankenhaus isoliert, daher spiegelt die hohe Hospitalisierungsquote hier eher eine infektionshygienische Maßnahme wider [11]. Dieses Vorgehen änderte sich zunehmend mit dem Anstieg der Fallzahlen und der steigenden Belastung der Krankenhauskapazitäten während der ersten Welle. Der im Vergleich zur zweiten Welle höhere Anteil schwerer und kritischer Fälle kann jedoch nur zum Teil auf die niedrigschwellige Hospitalisierung von COVID-19-Fällen zurückgeführt werden. Vielmehr ist von multiplen Einflüssen auszugehen.

Dabei ist beispielsweise die veränderte Teststrategie zu berücksichtigen. Zu Beginn der *Phase 1* befanden sich die Testkapazitäten noch im Auf- bzw. Ausbau [4, 25, 26]. Mit der Einführung von Reihentestungen (z. B. am Flughafen ab KW 31/2020) und der Verfügbarkeit von Antigenschnelltests in Krankenhäusern und Alten- und Pflegeheimen ab KW 42/2020 (*Phase 3*) wurden in Deutschland jedoch auch Fälle mit nur geringer Symptomatik (mit und ohne schweren Verlauf) in den Meldedaten gemäß IfSG besser erfasst. Dagegen wurden in der syndromischen Krankenhaus-Surveillance ICOSARI in allen Phasen nur schwer erkrankte COVID-19-Fälle mit respiratorischer Symptomatik (SARI) betrachtet. Damit sind in diesem System stabilere Aussagen zum Anteil kritischer Verläufe in den verschiedenen Phasen möglich, unabhängig von der gewählten Teststrategie und den Empfehlungen zur niedrigschwelligen Krankenhausaufnahme.

Dennoch zeigte sich auch im ICOSARI-System ein etwas höherer Anteil intensivpflichtiger und beatmeter COVID-SARI-Fälle, was mit Erfahrungen aus europäischen Ländern und den USA übereinstimmt [27]. Dies wird insbesondere auf verbesserte Behandlungskonzepte u. a. durch eine verstärkte Nutzung nichtmechanischer Beatmung in späteren Phasen (insbesondere *Phase 3*) zurückgeführt [28–30]. Zudem wurde in Deutschland während der zweiten Welle ein System zur Verlegung von intensivmedizinisch behandelten Patienten etabliert, um die Überlastung einzelner Krankenhäuser zu vermeiden (Kleeblattsystem; [31]). Dies zeigte sich im ICOSARI-System in der kürzeren Hospitalisierungsdauer von beatmeten COVID-SARI-Patienten mit einer späteren Verlegung. Ergänzend zu diesen Punkten haben möglichweise auch die ab KW 53/2020 eingeführten Impfungen im Abklingen der zweiten Welle zu einer verringerten Krankheitsschwere in der Altersgruppe ab 80 Jahren beigetragen [32].

Die Altersverteilung bei kritischen Krankheitsverläufen blieb in den *Phasen 1 bis 3* weitestgehend stabil. Während vor allem COVID-SARI-Fälle der Altersgruppe 60 bis 79 Jahre intensivmedizinisch behandelt oder beatmet wurden, war der Anteil verstorbener COVID-SARI-Fälle in der Altersgruppe ab 80 Jahre besonders hoch. Jedoch wurde deutlich, dass auch jüngere Altersgruppen von kritischen Verläufen betroffen waren: So wurde aus der Altersgruppe 35 bis 59 Jahre während der ersten und zweiten Welle jeder 3. hospitalisierte COVID-SARI-Patient intensivmedizinisch behandelt, jeder 5. wurde mechanisch beatmet und etwa jeder 20. COVID-SARI-Patient der Altersgruppe 35 bis 59 Jahre verstarb während des Krankenhausaufenthalts.

Insgesamt decken sich die Ergebnisse zur Altersverteilung mit den ersten Auswertungen zur Krankheitsschwere im Meldesystem bzw. unter hospitalisierten COVID-SARI-Fällen sowie mit Analysen im internationalen Vergleich [4, 11, 33–36]. So stellten auch Ioannidis et al. bei der Untersuchung von COVID-19-Todesfällen in Europa und den USA fest, dass sich die Altersverteilungen der ersten und zweiten COVID-19-Welle kaum unterschieden und Alter ein wesentlicher Risikofaktor für einen schweren Verlauf ist [34].

Unabhängig von den Phasen wurden weibliche und männliche Fälle in ähnlichem Umfang übermittelt. Insgesamt waren jedoch männliche Fälle häufiger von schweren Verläufen betroffen, was sich insbesondere unter den intensivpflichtigen und den beatmungspflichtigen COVID-SARI-Fällen im Krankenhaus-Sentinel ICOSARI widerspiegelte. Dies wird zum Teil auf geschlechtsspezifische Alterungsprozesse im Immunsystem zurückgeführt, auch wenn hier noch weitere Analysen erforderlich sind [37].

### Limitationen

Die vorliegende Auswertung basiert auf der Bewertung von Surveillance-Daten und unterliegt damit einigen Limitationen. Wie bereits vielfach beschrieben sind die Meldungen gemäß IfSG abhängig von den zugrunde liegenden Kapazitäten in Laboren, Gesundheitsämtern und Landesgesundheitsbehörden [2, 4] und damit anfällig für Verzögerungen. Durch den gewählten Datenstand (11.05.2021) wurde versucht, den Einfluss von Nachmeldungen – insbesondere zu schwer erkrankten Fällen – zu reduzieren. Darüber hinaus hängen die Meldungen gemäß IfSG auch von der Inanspruchnahme der Testmöglichkeiten durch die Bevölkerung und der in Deutschland angewendeten Teststrategie ab. Dennoch deutet der bereits zu Beginn relativ hohe Anteil milder Fälle auf eine sensitive Teststrategie und eine gute Erfassung des Gesamtgeschehens im Meldesystem hin [4, 25, 26].

Ausbruchssettings sind durch die Gesundheitsämter teilweise schwer zu erheben. Vollständigkeit und Qualität hängen zum einen von verfügbaren Kapazitäten für Ermittlungstätigkeiten vor Ort ab. Zum anderen ist es für Erkrankte aufgrund der zum Teil eher unspezifischen Symptomatik und eines möglicherweise schleichenden Beginns der Erkrankung nicht immer möglich, im Nachhinein den wahrscheinlichen Zeitpunkt der Infektion anzugeben [8]. Es kann nicht ausgeschlossen werden, dass insbesondere leicht zu ermittelnde Settings, wie bspw. der private Haushalt, aufgrund besserer Nachverfolgbarkeit häufiger ermittelt wurden. Bei der Erfassung in der Meldesoftware kann zudem nur zwischen vorgegebenen Kategorien ausgewählt werden. So fallen z. B. Ausbrüche in Fleisch verarbeitenden Betrieben unter das Setting Arbeitsplatz [8].

Im Rahmen der syndromischen Krankenhaus-Surveillance wurde eine etablierte SARI-Falldefinition durch ICD-10-Codes für eine laborbestätigte COVID-19-Erkrankung ergänzt [5, 6]. Zwar wurden im Frühling 2020 COVID-19-Fälle möglicherweise noch untererfasst. Aufgrund der bereits zu Beginn insgesamt gezielten Testung von schweren Fällen mit respiratorischer Symptomatik und des ab Juli systematischen Screenings von Neuaufnahmen in den ICOSARI-Krankenhäusern ist hier jedoch von einer eher guten Erfassung in ICOSARI auszugehen. Der Fokus auf ausschließlich COVID-19-Fälle mit einer schweren respiratorischen Symptomatik lässt zwar keine Aussagen zu Fällen ohne respiratorische Symptomatik zu. Im Gegensatz zu Meldungen gemäß IfSG erfolgt die Erfassung in ICOSARI jedoch unabhängig von der bundesweiten Teststrategie [2] und deckt sich daher gut mit den Ergebnissen anderer Analysen zu hospitalisierten COVID-19-Fällen und schweren Verläufen in Deutschland [28, 33, 38]. Darüber hinaus kann es jedoch bei hoher regionaler Belastung des Gesundheitssystems – insbesondere während der *Phase 3* – durch die Festlegung von Schwerpunktkliniken und innerdeutschen Verlegungen (Kleeblattkonzept) zu einer Verzerrung der Anzahl an COVID-19-Patienten im ICOSARI-System gekommen sein [31].

Mit dem Abklingen der zweiten Welle zu Beginn des Jahres 2021 stieg der Anteil der besorgniserregenden Virusvariante (Variant of Concern, VOC) B.1.1.7 in Deutschland, lag aber bis zur KW 8/2021 noch unter 50 % [39]. Es ist davon auszugehen, dass der Einfluss der VOC im Jahr 2020 eine untergeordnete Rolle spielte. Nicht ganz ausgeschlossen werden kann aber ein Einfluss auf einzelne Stadt- und Landkreise in Sachsen, die in *Phase 3* eine hohe Inzidenz aufwiesen. Diese lagen vor allem an der Grenze zur Tschechischen Republik, in der am Ende das Jahres 2020 bereits eine erhöhte Zirkulation von VOC (insbesondere B.1.1.7) vermutet wurde.

## Fazit

Die COVID-19-Pandemie in Deutschland war bis März 2021 durch verschiedene Phasen geprägt, die durch 2 unterschiedlich schwer verlaufende Wellen im Frühjahr und Herbst und eine Sommerplateauphase mit eher milden Verläufen charakterisiert waren. Im Vergleich zur ersten Welle erstreckte sich die zweite Welle über einen längeren Zeitraum, in dem wöchentlich deutlich mehr COVID-19-Fälle und COVID-SARI-Fälle in den verschiedenen Surveillance-Systemen beobachtet wurden. Mit Blick auf die Altersverteilung wurden in Phasen mit geringerer SARS-CoV-2-Aktivität (Phase sporadischer Fälle und Sommerplateau) eher jüngere und reiseassoziierte Fälle beobachtet, während dagegen in den beiden COVID-19-Wellen auch viele ältere Menschen erkrankten. Unabhängig von den Phasen waren jedoch vor allem ältere Menschen und im Verhältnis mehr Männer als Frauen von schweren bzw. kritischen Krankheitsverläufen betroffen.

Zusätzlich konnte gezeigt werden, dass sich die Meldedaten gemäß IfSG und die syndromische Surveillance gegenseitig ergänzen und auf diese Weise einen guten Überblick zum Gesamtgeschehen in Deutschland hinsichtlich Transmission und Krankheitsschwere geben können.
